# Effect of atorvastatin on iron metabolism regulation in patients with chronic kidney disease – a randomized double blind crossover study

**DOI:** 10.1080/0886022X.2018.1535983

**Published:** 2018-12-27

**Authors:** Anna Masajtis-Zagajewska, Michal Nowicki

**Affiliations:** Department of Nephrology, Hypertension and Kidney Transplantation, Medical University of Lodz, University Hospital and Teaching Center, Lodz, Poland

**Keywords:** Hepcidin, hemojuvelin, inflammation, iron metabolism, chronic kidney disease

## Abstract

**Introduction:** To determine the effect of 6-month administration of atorvastatin on hepcidin and hemojuvelin levels, inflammatory parameters and iron metabolism in patients with chronic kidney disease (CKD) stages 3 and 4.

**Methods:** Thirty six statin- and erythropoiesis-stimulating agent-naive patients with CKD stages 3 and 4 and LDL cholesterol ≥100 mg/dl received atorvastatin or placebo for two 6-month periods in a double blind, randomized crossover study. Hepcidin, hemojuvelin, hsCRP, IL-6, hemoglobin, red blood cell distribution width, iron, total iron binding capacity (TIBC), and unsaturated iron binding capacity (UIBC) were measured before and after each treatment period.

**Results:** Hepcidin decreased (from 102 [307] to 63 [170] pg/ml (*p* > .001)) in the course of statin therapy but remained unchanged after placebo administration (173 [256] to 153 [204] pg/ml, respectively). Hemojuvelin did not change after either part of the study. Both IL-6 and hsCRP decreased following statin therapy (from 8.7 [12.0] to 8.1 [13.9] pg/ml; *p* = .04 and from 4.7 [4.0] to 4.0 [3.6] mg/l; *p* = .4, respectively), but did not change after placebo administration. Blood hemoglobin increased slightly but significantly after 6-month statin therapy (from 11.6 ± 1.6 to 11.9 ± 1.5 g/dl, *p* = .002), and was unchanged after placebo treatment. TIBC and UIBC increased significantly after 6-month statin therapy, and serum iron also tended to increase. The change of eGFR during the study did not differ between the two treatment periods.

**Conclusions:** Statin may have a small but potentially beneficial effect on serum hepcidin, which may lead to improvement of anemia control in CKD patients.

## Introduction

Chronic kidney disease (CKD) is characterized by renal anemia caused by a relative deficiency of endogenous erythropoietin secretion, increased blood loss, and shortened red blood cell survival. CKD is also accompanied by mild to moderate chronic inflammation which may result in decreased availability of iron, and thereby contribute to the worsening of renal anemia [1]. Hepcidin is an endogenous antimicrobial peptide secreted by the liver [2,3]. Hepcidin serves as a critical determinant of iron metabolism, inhibiting intestinal absorption of dietary iron, release of iron from macrophages, and transfer of iron stored in hepatocytes. Hepcidin serves also as an acute phase protein and its expression is induced by inflammation [4]. Serum hepcidin is increased in inflammatory states and in CKD, leading to decreased release of iron from macrophages resulting in insufficient erythropoiesis and anemia [5]. Animal studies have also shown that increased expression of hepcidin leads to a blunted erythropoietic response to endogenous epoetin [6]. Hepcidin degrades ferroportin, which leads to accumulation of iron in macrophages and hepatocytes [7,8]. However, despite adequate deposits of iron, the available amount of circulating hemoglobin could be reduced.

The hepcidin level is inversely correlated with the estimated glomerular filtration rate in patients with CKD, and its level is reduced during epoetin administration [9]. Serum hepcidin concentration in patients with CKD increases progressively with the severity of CKD and can only be partly reduced by hemodialysis in end-stage kidney disease [10–14]. It has been recently postulated that the increased production of hepcidin may be an important cause of resistance to erythropoietin stimulating agent (ESA) therapy in advanced or end-stage CKD [6].

Hepcidin expression is regulated through the bone morphogenetic protein-hemojuvelin (BMP-HJV) signaling pathway. Hemojuvelin is a member of the repulsive guidance molecule family and appeared lately to be a key regulator of iron-dependent secretion of hepcidin. This iron-regulatory protein is mainly expressed in the liver, skeletal muscle, and heart and its production follows a specific process. The furin mRNA level is negatively regulated by iron concentrations [15,16]. The HJV produced by furin cleavage downregulates hepcidin expression by competing with hepatocyte membrane-bound mHJV for BMP binding. When not inhibited by HJV, this binding starts a cascade ending with an active protein complex that translocates into the nucleus and regulates hepcidin expression positively [17,18]. The inhibitors of 3-hydroxy-3-methy-glutaryl-co-enzyme A reductase reduce serum lipid levels and may have several other effects, including reduction of inflammation. Since statin therapy may overcome ESA hyporesponsiveness [19], it might be postulated that the beneficial effect of statins on response to ESA therapy may be mediated by a decreased release of inflammatory mediators and increased release of iron from its stores [19,20]. This concept is supported by the results of the study which shows that administration of fluvastatin in patients with end-stage kidney disease leads to a decrease of prohepcidin, a prohormone of hepcidin [21].

To the best of our knowledge, there have been no studies on the effect of statins on hepcidin and hemojuvelin levels, and thus iron metabolism and anemia in ESA naive patients with CKD not yet on dialysis.

The aim of the study was to assess the effect of 6-month administration of atorvastatin on hepcidin and hemojuvelin levels, inflammatory parameters and iron metabolism in patients with stages 3 and 4 CKD.

## Materials and methods

We obtained permission to perform this study from the Local Ethics Committee and followed the guidelines of the Helsinki Declaration. All the subjects were informed about the aims and design of the study, and provided a written informed consent prior to participation. The study was funded by the Medical University of Lodz research Grant No. 503/1-151-02/503-01.

### Patients and study design

**Figure 1.**
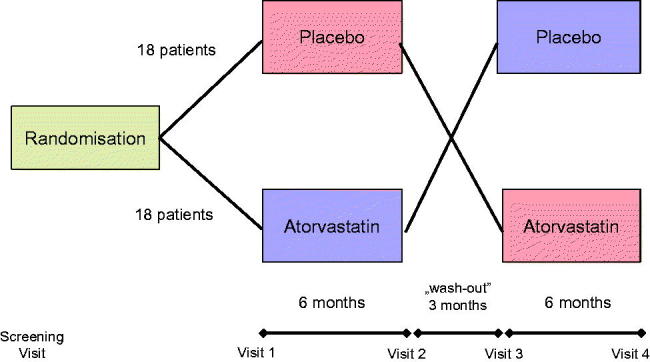
Study design.

In a double-blind placebo-controlled crossover study, the patients randomly received either atorvastatin (20 mg in a single evening dose) or identically looking placebo tablets for the first 6 months. The treatment arms cross-changed after the wash-out period and the therapy with placebo or atorvastatin continued for another 6 months. The measurements were taken four times, i.e., prior to and at the end of each treatment period (Figure 1).

The patients were included in the study if they had a stable kidney function (±5%) for at least 6 months prior to the study and were not expected to start the renal replacement therapy within the next 6 months. All the patients were ESAs naïve, with adequate iron stores (ferritin level above >150 ng/ml and TSAT above 20%), with a constant dose of oral iron administered for at least the last 6 months, and not receiving any blood transfusion or intravenous iron for 3 months before the study. The doses of all other medications were unchanged throughout the study except for emergencies.

Exclusion criteria included the hemoglobin level below 10 g/dl, uncontrolled or treatment-resistant hypertension [22], persistent urine protein excretion >1 g/24 h, or chronic steroid therapy with a dose of prednisone >5 mg/24 h, chronic heart failure NYHA stage 3 or higher, malignancy, administration of any lipid-lowering medication for 6 months prior to the study and administration of oral phosphate binders.

Blood samples were obtained after overnight fasting, collected in EDTA tubes, and promptly centrifuged at 2000×*g* at 2–8 °C for 10 min within 30 min of sampling. The plasma was stored in aliquots at −70 °C. The following biochemical parameters were measured at each visit: serum creatinine, total-, HDL-, LDL-cholesterol, triglyceride, uric acid, total protein, albumin, hepcidin, hemojuvelin, iron, ferritin, total iron binding capacity (TIBC), unsaturated iron binding capacity (UIBC), hemoglobin, red blood cell distribution width (RDW), C-reactive protein, as well as alanine and aspartate aminotransferase.

Plasma hemojuvelin and hepcidin concentrations were measured using a commercial immunoassay from USCN Life Science (Houston, TX). All the other parameters were measured with standard laboratory automated methods in the local laboratory. TIBC values were calculated from serum iron and UIBC, both of which were measured with colorimetric methods. RDW-CV calculation was based on both the width of the distribution curve and the mean cell size. It was calculated by an automated hematology analyzer by dividing the standard deviation of the mean cell size by the MCV of the red cells and multiplying by 100 to convert to a percentage.

The results are expressed as mean ± SD or median ± interquartile ratio (IQR) dependent on the normality of variable distribution. Statistical significance was defined as *p*<.05. The normality of data was checked by the Kolmogorov–Smirnov test. The paired *t*-test was used to analyze the differences between statin and placebo. In the case of non-normally distributed variables, the Wilcoxon test was used. Pearson’s correlation coefficient was calculated to assess the associations between the variables. Statistical analysis was performed using Statistica for Windows (version 10PL, StatSoft, Tulsa, OK).

## Results

The participants were selected from the population treated in the outpatient clinic of the tertiary nephrology center. After prescreening, 60 potentially illegible patients were identified in our database. After screening, 36 patients were included and all of them completed the study visits according to the protocol. The study population comprised 19 women and 17 men, mean age 56 ± 11 years with CKD stages 3 and 4. The clinical characteristics of the study population are presented in Table 1. The causes of CKD included chronic glomerular disease in 12 patients, diabetic kidney disease in 8, adult polycystic kidney disease in 4, arterial hypertension in 4, tubulointerstitial nephritis in 1, and unknown causes in seven patients. Eight patients suffered from type 2 diabetes, arterial hypertension was diagnosed in 34 patients, and ischemic heart disease in five patients. No clinically important side-effects of statin therapy were observed with the exception of a transient mild muscle pain in one of the patients at the beginning of atorvastatin therapy during the study. During the entire study periods, no acute illnesses including infections of such severity that in the opinion of the investigators could affect the course of the study were observed at scheduled visits.

**Table 1.** Baseline clinical and biochemical characteristics of the study group.

	Mean ± SD	Median [IQR]	Range
Age (years)	57 ± 10	59 [15.7]	33–70
BMI (kg/m^2^)	25.9 ± 4.5	25.4 [5.9]	17.8–34.8
Creatinine (mg/dl)	2.8 ± 0.8	2.3 [0.2]	1.41–3.87
Estimated GFR (ml/min/1.73 m^3^)^a^	22.7 ± 9.4	17.4 [13.1]	15.3–47.3
Hemoglobin (g/dl)	11.6 ± 1.6	11.5 [2.5]	9.1–14.7
Total cholesterol (mg/dl)	232.9 ± 47.6	225 [50]	154–367
HDL-cholesterol (mg/dl)	56.9 ± 16.7	56 [28.2]	32–89
LDL-cholesterol (mg/dl)	146 ± 29	146 [28.3]	72–201
Triglycerides (mg/dl)	170 ± 60	163 [46.7]	68–297
Uric acid (mg/dl)	7.5 ± 1.2	7.4 [2.1]	5–9.7
Total protein (g/l)	61.5 ± 8.7	63.9 [13.2]	50.2–75.4
Albumin (g/l)	37.8 ± 6.8	37.7 [9.3]	26.1–54.9
Iron (μg/dl)	69.1 ± 19.4	67 [31.5]	37–99
Ferritin (ng/ml)	177 ± 22.8	173 [19]	151–252
UIBC (μg/dl)	187 ± 49	195 [61]	64–296
TSAT (%)	25.3 ± 4.8	24 [5.7]	20–39
TIBC (μg/dl)	255 ± 43	262 [57]	131–335
Serum CRP (mg/l)	5.1 ± 2.9	4.7 [4.0]	0.27–9.87
AspAT (U/L)	25.4 ± 10.9	24.5 [10.7]	9–59
AlAT (U/L)	29.4 ± 12.4	31 [17]	12–34
Serum hepcidin (pg/ml)	244 ± 334	102 [307]	22.3–546.8
Serum hemojuvelin (ng/ml)	36.7 ± 14.3	37.6 [14.3]	14.9–80.3
Serum IL-6 (pg/ml)	15.1 ± 16.5	8.7 [12.0]	0.23–40.75

Results are expressed as mean ± SD [median (IQR)].

^a^eGFR: estimated glomerular filtration rates – calculated using the MDRD abbreviated formula; UIBC: unsaturated iron binding capacity; TSAT: transferrin saturation; TIBC: total iron binding capacity; CRP: C-reactive protein; AspAT: aspartate aminotransferases; AlAT: alanine aminotransferases; IL-6: interleukin-6.

Table 1 shows the clinical and biochemical characteristics of the participants.

The effects of atorvastatin and placebo on serum concentration of hepcidin, hemojuvelin, hemoglobin, and iron metabolism, i.e., serum iron, ferritin, TSAT, TIBC, UIBC, red cell distribution width (RDW), are shown in Table 2. There was a small but significant increase of serum hemoglobin in the course of atorvastatin administration (from 11.6 ± 1.6 g/dl to 11.9 ± 1.5 g/dl; *p* = .002). Similar effects were also observed in the case of UIBC and TIBC. There was a significant decrease of serum hepcidin concentration at the end of statin therapy compared to the baseline. All these parameters did not change significantly during placebo administration. Serum ferritin, TSAT, RDW, and hemojuvelin concentration did not change in either part of the study.

**Table 2.** Hematological and inflammatory parameters and kidney function during the study.

	Before statin	After statin	Δ after–before statin 95% CI	*p* Value	Before placebo	After placebo	Δ after–before placebo 95% Cl	*p* Value	Δ Statin/placebo 95% CI	Statin/placebo therapy *p* value
Hemoglobin (g/dl)	11.6 ± 1.6	11.9 ± 1.5	0.24 ± 0.44 (95% CI: 0.1–0.4)	.002	11.7 ± 1.3	11.8 ± 1.3	0.18 ± 0.89 (95% CI: –0.12 to 0.48)	.2	0.06 ± 0.9 (95% CI: –0.24 to 0.36)	.7
Iron (μg/dl)	67 [31.5]	68 [30.7]	3.2 ± 12.4 (95% CI: –0.9 to 7.3)	.2	68.5 [23.5]	67 [18]	2.7 ± 22.5 (95% CI: –4.8 to 10.2)	.9	0 ± 9.2 (95% CI: –3.09 to 3.09)	.4
Ferritin (ng/ml)	173 [19]	140 [65]	–3.19 ± 77.2 (95% CI: –29.1 to 22.7)	.06	163 [19]	157 [51]	–2.5 ± 50.3 (95% CI: 19.4–14.4)	.7	–0.68 ± 64.3 (95% CI: 22.3–20.9)	.2
UIBC (μg/dl)	187 ± 49	198 ± 50	11.3 ± 28.2 (95% CI: 1.9–20.7)	.02	205 ± 53	201 ± 44	–4.1 ± 39.2 (95% CI: –17.2 to 9)	.5	15.3 ± 47.9 (95% CI: –0.8 to 31.4)	.07
TSAT (%)	24 [5.7]	25.5 [6.7]	0.14 ± 5.6 (95% CI: –1.66 to 1.94)	.6	25 [7.5]	25 [5.7]	0.4 ± 4.6 (95% CI: –1.14 to 1.94)	.8	0.3 ± 7.4 (95% CI: –2.1 to 2.7)	.9
TIBC (μg/dl)	267 [57]	272 [96]	13.1 ± 26.1 (95% CI: 4.4–21.8)	.04	272 ± 49	270 ± 44	–1.6 ± 32.5 (95% CI –12.5 to 9.3)	.7	14.8 ± 44.7 (95% Cl 0.2–29.8)	.8
Hepcidin (pg/ml)	102 [307]	63 [170]	–85 ± 188.7 (95% Cl –148.4 to 21.6)	>.001	173 [256]	153 [204]	–21.1 ± 137.6 (95% Cl 67.3–25.1)	.9	–63.9 ± 171.8 (95% Cl 121.6–6.2)	.005
Hemojuvelin (ng/ml)	37.6 [14.3]	40.1 [10.6]	0.7 ± 10.8 (95% Cl –2.9 to 4.3)	.8	35.5 ± 10.5	35.4 ± 10.1	–1.2 ± 7.1 (95% Cl 1.1–5.9)	.3	1.95 ± 13.2 (95% Cl –2.4 to 6.3)	.4
RDW (%)	14.6 ± 1.3	14.3 ± 1.3	–0.35 ± 1.3 (95% Cl –0.75 to 0.05)	.6	14.3 [1.4]]	14.2 [1.7]	0.06 ± 1.1 (95% Cl –0.3 to 0.42)	.8	–0.4 ± 1.7 (95% Cl –0.9 to 0.1)	.8
MCH (pg)	27.9 [1.1]	28.6 [1.0]	0.4 ± 0.8 (95% Cl 0.14–0.66)	.01	28.5 [1.1]	28.4 [1.0]	0.16 ± 1 (95% Cl –0.14 to 0.46)	.5	0.22 ± 1.2 (95% Cl –0.18 to 0.62)	.2
MCHC (g/dl)	32.7 ± 1.5	33.1 ± 1.5	0.3 ± 1 (95% Cl 0–0.6)	.04	33.0 [3.2]	33.2 [3.2]	–0.2 ± 1.2 (95% Cl –0.6 to 0.2)	.4	0.5 ± 1.5 (95% Cl 0–1.0)	.08
hsCRP (mg/l)	4.7 [4.0]	4.0 [3.6]	–0.5 ± 1.5 (95% Cl 1.0–0)	.4	4.5 ± 2.3	4.5 ± 1.8	0.01 ± 0.9 (95% Cl 0.29–0.31)	.9	–0.5 ± 1.7 (95% Cl 1.0–0)	.06
IL-6 (pg/ml)	8.7 [12.0]	8.1 [13.9]	2.6 ± 7.4 (95% Cl 0.2–5.0)	.04	7.4 [9.4]	6.3 [6.6]	–2.5 ± 8.9 (95% Cl –5.4 to 0.4)	.4	–0.1 ± 5.5 (95% Cl 1.9–1.7)	.9
eGFR (ml/min/1.73 m^3^)	17.4 [13.1]	17.3 [10.8]	–1.6 ± 3.7 (95% Cl –2.8 to 0.6)	.05	17.8 [11.2]	17.4 [9.2]	–0.9 ± 1.8 (95% Cl –1.5 to –0.3)	.03	0.7 ± 3.9 (95% Cl –0.6 to 2.0)	.5

Results are expressed as mean ± SD in the case of non-normally distributed variables or [median (IQR)] in the case of non-normally distributed variables.

eGFR: estimated glomerular filtration rates – calculated using the MDRD abbreviated formula; UIBC: unsaturated iron binding capacity; TSAT: transferrin saturation; TIBC: total iron binding capacity; IL-6: interleukin-6.

**Figure 2.**
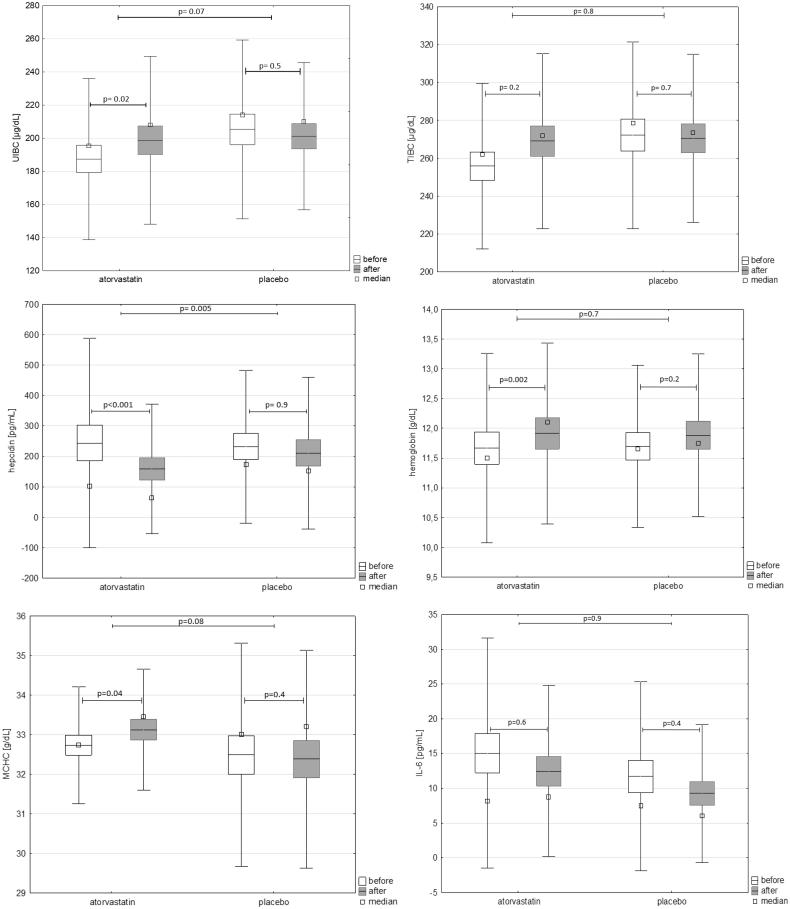
Changes of UIBC, TIBC, hepcidin, hemoglobin, MCHC, and IL-6 after placebo and atorvastatin treatment.

Kidney function (eGFR) significantly decreased in both study periods (Table 2), i.e., during administration of atorvastatin (from 22.7 ± 9.4 to 21.0 ± 7.9 ml/min/1.73 m^3^, *p* = .01) and during administration of placebo (22.1 ± 8.9 to 21.1 ± 8.7, *p* = .005). There was no significant difference between mean changes of eGFR between both periods of the study (*p* = .3). Table 2 shows also the inflammation parameters assessed during the study. Both hsCRP and IL-6 decreased significantly in the course of treatment with atorvastatin, but remained unchanged during the placebo treatment period. Figure 2 shows the changes of UIBC, TIBC, hepcidin, hemoglobin, MCHC, and IL-6 after placebo and atorvastatin treatment.

Total cholesterol, LDL-cholesterol, triglyceride, and uric acid decreased significantly and HDL-cholesterol increased significantly after atorvastatin therapy (Table 3). These parameters did not significantly change after placebo treatment. Serum concentration of alanine and aspartate aminotransferase increased significantly after atorvastatin but not after placebo therapy (Table 3).

**Table 3.** Serum concentration of cholesterol, HDL, LDL, triglycerides, uric acid, as well as aspartate and alanine aminotransferase during the study.

	Before statin	After statin	Δ after–before statin 95% CI	*p* Value	Before placebo	After placebo	Δ after–before placebo 95% CI	*p* Value	Δ Statin/placebo 95% CI	Statin/placebo therapy *p* value
Total cholesterol (mg/dl)	225.5 [50]	195 [41]	–34.0 ± 32.8 (95% CI –45 to –23)	<.001	218 [55]	212 [49]	–5.3 ± 24.1 (95% CI –13.4 to 2.8)	.9	–28.6 ± 37.8 (95% CI –41.3 to –15.9)	<.001
HDL-cholesterol (mg/dl)	56.9 ± 16.7	63.7 ± 17.9	6.7 ± 12.5 (95% CI 2.5–10.9)	.003	56 [27]	56 [31.3]	0.6 ± 10.8 (95% CI –3.0 to 4.2	.8	6.1 ± 13.3 (95% CI 1.7–10.5)	.04
LDL-cholesterol (mg/dl)	146 ± 28	107 ± 26	–38.4 ± 29.1 (95% CI –48.2 to –28.6)	<.001	146 [24]	144 [40]	–5.2 ± 20.2 (95% CI –11.9 to 1.5)	.9	–32.3 ± 31.0 (95% CI –42.7 to –21.9)	<.001
Triglyceride (mg/dl)	165 [36]	145 [43]	–23.8 ± 36.9 (95% CI 36.2 to –11.4)	.02	154 [25]	149 [31]	–5.3 ± 17.8 (95% CI –11.2 to 0.6)	.03	–18.5 ± 36.9 (95% CI –30.8 to –6.1)	.01
Uric acid (mg/dl)	7.5 ± 1.3	7.1 ± 1.2	–0.4 ± 0.9 (95% CI –0.7 to 0.1)	.01	7.2 ± 1.4	7.1 ± 1.2	–0.2 ± 0.8 (95% CI –0.4 to 0)	.1	–0.2 ± 1.2 (95% CI –0.6 to 1.0)	.3
AspAT (U/L)	24.5 [10.7]	28 [11.7]	4.5 ± 11.8 (95% CI 2.1–6.9)	.003	21 [6.7]	21 [9]	1.1 ± 5.1 (95% CI –0.6 to 2.8)	.1	3.4 ± 13.5 (95% CI –1.1 to 7.9)	.3
AlAT (U/L)	29.4 ± 12.5	32.3 ± 12.5	2.1 ± 7.8 (95% CI –0.5 to 4.7)	.04	26.1 ± 8.9	27.1 ± 9.4	0,8 ± 2.7 (95% CI –0.1 to 1.7)	.09	–2.9 ± 9.2 (95% CI –5.9 to 0.1)	.2

Results are expressed as mean ± SD in the case of non-normally distributed variables or [median (IQR)] in the case of non-normally distributed variables.

AspAT: aspartate aminotransferase; AlAT: alanine aminotransferase.

No significant correlations were found between serum hepcidin, IL-6 and ferritin at baseline (*r* = .37, *p* = .9; *r* = .058, *p* = .7, respectively) and between the changes of serum hepcidin, IL-6 and ferritin after atorvastatin therapy (*r* = –0.316, *p* = .06; *r* = .006, *p* = .9, respectively) and after placebo treatment (*r* = –0.32, *p* = .06; *r* = .01, *p* = .9, respectively). There was a significant positive correlation between serum hepcidin and hsCRP (*r* = 0.37; *p* = .025) at baseline. The changes of serum hepcidin and changes of hsCRP during atorvastatin therapy (*r* = .13, *p* = .4) and placebo administration (*r* = .022, *p* = .2) did not correlate significantly.

## Discussion

A chronic inflammatory state may be an additional factor contributing to increased expression of acute phase proteins such as hepcidin and ferritin. There are two major patterns of acute-phase responses in hepatocytes [23,24]. Type 1 response is induced by IL-1-like cytokines, whereas type 2 response is induced by IL-6. Although some studies showed a significant correlation between serum hepcidin and ferritin in patients with different disorders affecting iron metabolism [25,26], the pathways regulating their expression seem to be distinct. The conflicting findings may also result from different methods of measurement, since Ashby et al. [9] used a radioimmunoassay, while we used an enzyme-linked immunoassay.

It is of note that we revealed a significant positive correlation between hepcidin and CRP at baseline, while no relation was found in the case of IL-6. That finding was consistent with the results of van der Weerd et al. [27], though that study was conducted in patients who were already on chronic HD. Some other studies also reported significant positive correlations between serum hepcidin and CRP or IL-6 in CKD patients as well as in subjects with fully preserved renal function [3,28,29]. Zaritsky et al. [3] found a significant correlation between serum hsCRP and hepcidin at all stages of CKD. Their finding may provide the evidence for the existence of a pathophysiological connection between hepcidin and CRP. However, our findings are not concordant with the findings of Ashby et al. [9], who did not find any relation between serum hepcidin and inflammatory markers (hsCRP and IL-6) in both CKD and HD patients. However, this may be due to the use of a different assay to measure serum hepcidin.

The relation between serum hepcidin and ferritin has been well established. Serum ferritin functions as a biomarker of both iron stores in the reticulo-endothelial system and as an acute phase protein. The same applies to serum hepcidin. Therefore, these two markers are expected to change in parallel. Interestingly, we did not observe any significant change of serum ferritin after statin treatment, while serum hepcidin decreased significantly. We also did not find any correlation between the changes of serum hepcidin and ferritin during statin therapy. Our findings contrast with the results of the study of Ashby et al. [9], who reported a significant correlation between serum hepcidin and ferritin in CKD patients not yet on dialysis but not in chronic hemodialysis patients. However, CKD patients in their study [9] had a wide range of renal function impairment with estimated GFR 8–98 ml/min, while in our study CKD patients were at more advanced stages of kidney disease.

Renal anemia is undoubtedly a multifactorial condition. Its treatment is multidirectional due to complex interactions between chronic inflammation, iron metabolism and relative or absolute erythropoietin deficiency. Our study was designed to explore the potential benefit of statin in the management of anemia in patients with CKD. We postulated that the effect of statin on blood hemoglobin might be mediated by its anti-inflammatory properties. The present study was cross-over randomized double-blind to allow a direct comparison of the effect of statin and placebo within the same group of patients.

The effects of statin therapy on the inflammatory parameters observed in our patients are in agreement with several other studies carried out in CKD patients. The results of the seminal Study of Heart and Renal Protection (SHARP) encompassing 9270 patients with CKD (3023 on dialysis and 6247 not) showed that the lowering of LDL cholesterol with a combination of simvastatin and ezetimibe reduced the risk of major atherosclerotic events in CKD patients with different degrees of renal function impairment [30]. In other studies that focused on CKD stages 3 and 4, statins also proved their lipid-lowering and anti-inflammatory effect [31–33]. Panichi et al. [34] found that 6 months of simvastatin therapy with a daily dose of 40 mg in stages 3 and 4 CKD caused a significant decrease of serum inflammatory markers CRP and IL-6. Similarly, in the study of Goicoechea et al. [33], 6 months of atorvastatin administration at the same dose of 20 mg/day as in our study led to a significant decrease of CRP, TNF-α, and IL-1β. In another study, the most potent HMG-CoA reductase inhibitor rosuvastatin induced a similar anti-inflammatory effect in CKD patients [32]. Our study showed only a tendency of serum inflammatory markers such as CRP and IL-6 to decrease after statin therapy, and this effect was not significant in comparison with changes of these parameters after placebo therapy.

Although HMG-CoA reductase inhibitors are considered generally safe in early CKD, there are differences in their pharmacokinetic properties that might determine the choice of a particular statin in advanced CKD or in end-stage renal disease. We have chosen atorvastatin for our study because it has negligible (<2%) renal elimination [35] and does not require any dose adjustment even in patients with eGFR below 30 ml/min/1.73 m^2^. We have selected a mid-range dose of atorvastatin since our patients had only moderate serum lipid abnormalities and the same dose was used in earlier studies in CKD [33].

Both after statin and placebo therapy, we observed a deterioration of eGFR, there was, however, no significant difference between mean changes of eGFR in both periods of the study. Our findings corroborate the results of previous studies including the largest SHARP trial [30] that showed no effect of statin on kidney disease progression. The effect may be however dependent on a dose of statin. One recent metaanalysis [36] showed that high-intensity statin therapy slightly slowed a decline in eGFR in population with CKD compared with control, but moderate- and low-intensity statins, i.e., 10–20 mg/24 h atorvastatin, did not. We used moderate-intensity statin and that therapy was not found to have any significant effect on eGFR compared to placebo.

In order to exclude the effect of ESA on iron metabolism, all the patients in our study were ESA naive with the baseline hemoglobin concentration over 10 g/dl, with adequate iron stores, and with a constant dose of oral iron therapy for at least the last 6 months. We therefore postulate that the increase of hemoglobin during administration of atorvastatin with a significant increase of TIBC and UIBC may be most likely explained in our study by the anti-inflammatory action of statin leading to decreased expression of hepcidin. The fact that a change of eGFR, which is a major determinant of renal anemia, was similar after atorvastatin and placebo in our patients may confirm the concept.

The RDW reflects the variability in size of circulating red blood cells and is routinely reported by automated blood counts in laboratories, though it is rarely interpreted by physicians. Most studies have shown that RDW increases in the presence of systemic inflammation [37–39]. Moreover, RDW has been strongly associated with increased serum inflammatory markers and disease activity in patients with systemic lupus erythematosus [37,39] and with inflammatory bowel disease [39,40]. In a study involving almost 5000 outpatients [41], a significant association of increased RDW with low HDL-C and high levels of TG was reported. In our study, we did not find any significant changes of RDW after statin treatment, despite a consistent lipid-lowering effect. These results are consistent with the study of Kucera et al. [42], in which a higher dose of atorvastatin 40 mg/24 h was used and the time of the treatment was two times shorter than in our study. Akin et al. [43] also did not find any effect of 10–80 mg per day of atorvastatin on RDW.

Additionally, we observed a significant decrease of serum uric acid after statin. This observation is consistent with the study of Milionis et al. [44], who studied the effect of atorvastatin and simvastatin on uric acid homeostasis in patients treated for primary hyperlipidemia. Interestingly, in that study only atorvastatin, but not simvastatin, had a significant effect on serum uric acid [44].

The main limitation of our exploratory research was a relatively small group of patients and a possible interference of the progression of CKD on the parameters of anemia and iron metabolism during the study; however, the cross-over design of the study and randomization allowed intra-individual comparison of the effects of an active therapy and placebo. Due to the design of the study that including a long washout period that exceeded several times T1/2 of the drug the risk of carry‐over effect has been minimized. Since the subject clinical characteristics did not change during the whole duration of the study and none of the patients suffered from new or worsening diseases or underwent acute clinically relevant infections during the study, the interference of the period effect on the results was also eliminated. Additionally, we did not investigate the effect of atorvastatin on blood pressure and proteinuria, and we did not compare effects of different statins. To the best of our knowledge, the present study is the first to investigate the effect of statin therapy on hepcidin and hemojuvelin serum concentration, iron utilization and metabolism in ESA naive patients with moderate to advanced CKD.

## Conclusions

Treatment with statin may have potentially beneficial effects on the hepcidin level, and thus on iron metabolism in this population. Although the lipid-lowering effect of atorvastatin was only moderate, it may also have a potentially beneficial effect on hepcidin serum concentration, and thus on the inflammatory state, iron utilization and anemia in patients with CKD.
